# University of Virginia

**Published:** 1842-09

**Authors:** 


### UNIVERSITY OF VIRGINIA.—LATIN DIPLOMAS.

Our contemporary of the Boston Medical and Surgical Journal, notwithstanding the deplorable error in his "Natural History of Fishes, &c.", and notwithstanding, too, his excessive irritability (an infirmity which in his case might be called *touchiness*), brings to light a good thing now and then. Witness some remarks on the University of Virginia, in his Journal for August 10, from which we make the following extract.

"But there is one feature in the character of this school, so unlike that of any other amongst us, and yet so much in advance of them all in one particular respect, that not to proclaim it loudly, would do the institution injustice. It is, that 'any person of approved moral conduct may offer as a candidate for graduation, and receive the degree of M. D., without reference to the time he has been engaged in the study of medicine, or of joining the school, provided he undergoes, in a satisfactory manner, the various examinations prescribed by the enactments.' Here is an actual advancement in the mode of conferring degrees, which might be copied in other institutions, to the manifest advantage of society and the general interest of the institutions themselves. The faculty does not inquire of the candidate for their honors, whether he has studied five years or five days; but simply examines him in all that appertains to the knowledge which the statutes of the Legislature and the University declare to be the full measure of a doctor in medicine. If he sustains himself in the trial, he is at once presented with a testimonial of his attainments. Either this natural and philosophical system—which is essentially a common-sense affair —must ultimately be imitated by neighboring colleges, or we imagine the current will certainly by-and-by set in the direction of Virginia."

The feature here spoken of is entirely new to us, and possibly to most of our readers. We must give it our hearty approval. It seems perfectly consistent with that broad and liberal spirit that should characterise *all* institutions in this country. Our medical schools, in some of their regulations, are copied too closely after those of the old world, and unfortunately their models are far behind the genius of the age. If we make them, as we certainly do in some measure, the avenue to distinction, their approach should be left free and unobstructed. There is no reason why one, who, by his own unaided toil, in silence and seclusion, has qualified himself for the honors of the doctorate, should be denied them. In truth, the *reason* in the case is all on the other side.

Another matter, "essentially a common-sense affair" too, brought to mind whilst reading the article from which we have just quoted, may be mentioned here, although the connection does not seem very close. We allude to the *Latin* diplomas still retained by all of our colleges whether medical or literary; in some the degrees are even *conferred* in the Latin language. The custom is an old and time-honored one, it is true, but it has long survived the necessity which gave it birth, if such was, indeed, its origin. We had almost said, despite the seeming contradiction, that it is a relic of barbarity, and in reference to the barbarous Latin in which the diploma is generally couched, the expression might be allowed. If ever there was a good argument in favor of this custom, there certainly is none now. In many, if not most cases, it amounts to a positive absurdity. Many times the parchment is about as intelligible to the young man receiving it, as if filled with so much Choctaw or Pottawatomie. A medical friend with whom we were recently conversing on this subject informed us that when he was in Philadelphia, several years since, it was a very common thing for the medical graduates of the University of Pennsylvania to be running about the city to the various scholars of their acquaintance in order to procure a translation of their diploma. He heard and witnessed several such applications to the late Dr. Godman, who stated that it was an annual occurrence, and that the young men gave as a reason for so doing, the mortification they should experience to return home with a testimonial that they could not read. No doubt similar occurrences take place at every medical school in the country. Could any thing more forcibly demonstrate the utter absurdity of the whole proceeding? Let us not be understood as running a tilt against classical learning; that is a very different affair. No one feels a greater reverence for what are called the classics than ourselves. At the same time, however, this does not blind us to the claims of our mother-tongue; and we hope to see the day when both British and American colleges will employ it in the formulæ of their diplomas.

